# Hepatitis B virus X mediates podocyte pyroptosis by regulating the ROS/NLRP3 signaling pathway in hepatitis B virus-associated glomerulonephritis

**DOI:** 10.22038/IJBMS.2022.61105.13520

**Published:** 2022-01

**Authors:** Yani Yu, Hui Dong, Jingyi Sun, Baoshuang Li, Yueqi Chen, Moxuan Feng, Xiaoqian Yang, Wei Jiang

**Affiliations:** 1 Department of Nephrology, The Affiliated Hospital of Qingdao University, Qingdao 266003, Shandong, China; 2 Health Management Center, The Affiliated Hospital of Qingdao University, Qingdao 266003, Shandong, China; # These authors contributed equally to this work

**Keywords:** Glomerulonephritis, HBx, Nucleotide-binding - oligomerization domain-like receptor protein 3, Pyroptosis, Reactive oxygen species

## Abstract

**Objective(s):**

This study was designed to investigate whether HBx-induced podocyte injury is related to the nucleotide-binding oligomerization domain-like receptor protein 3 (NLRP3) inflammasome and the specific mechanism of the oxidative stress pathway in hepatitis B virus-associated glomerulonephritis (HBV-GN).

**Materials and Methods:**

The HBx gene was overexpressed in renal podocytes to mimic HBV-GN. Podocyte morphology was observed under a scanning electron microscope. Reactive oxygen species (ROS) generation was detected by dichlorodihydrofluorescein diacetate (DCFH-DA) assay. The podocytes in each group were treated with Hoechst 33342 and subjected to immunofluorescence staining. Caspase-1 activity and LDH levels were assessed with a Caspase-1 Activity Assay Kit and an LDH ELISA Kit, respectively. The expression of all pyroptosis-related proteins was examined by Western blot analysis.

**Results:**

Pyroptosis-related proteins, including NLRP3, apoptosis-associated speck-like protein containing card (ASC), caspase-1, IL-1β, and IL-18 (*P*<0.05), were up-regulated upon HBx overexpression, and caspase-1 enzyme activity and LDH and Desmin expression were also enhanced (*P*<0.05). NLRP3 knockdown attenuated the increased expression of pyroptosis-related proteins upon HBx overexpression (*P*<0.05), which was also achieved by the addition of an ROS inhibitor (*P*<0.05).

**Conclusion:**

HBx regulates podocyte pyroptosis in HBV-GN by targeting the NLRP3 inflammasome, and mitochondrial oxidative stress plays an important role in this process.

## Introduction

Hepatitis B virus (HBV), a member of the viral hepatitis family, can lead to chronic disease. HBV infection is also one of the most important factors in many other diseases (1). In addition to hepatitis, HBV often causes renal inflammation. HBV-associated glomerulonephritis (HBV-GN) is a type of kidney disease caused by deposition of immune complexes formed by binding of HBV to corresponding antibodies within glomeruli. Some scholars have suggested that the direct invasion of the kidney by HBV in this disease cannot be excluded (2). In previous studies, HBV-GN was proven to be related to deposition of the HBV antigen complex in the glomerulus, causing immune damage, direct infection of renal cells by the virus, and autoimmune pathogenesis due to HBV infection. However, the relationship between HBV and nephritis in the pathogenesis of this disease remains unclear and has not been comprehensively examined.

The nucleotide-binding oligomerization domain-like receptor protein 3 (NLRP3) inflammasome belongs to the NLR family and is composed of NLRP3, apoptosis-associated speck-like protein containing card (ASC), and procaspase-1. A key molecular platform of the innate immune system, the NLRP3 inflammasome regulates maturation of the proinflammatory cytokines IL-1β and IL-18 in response to microbial infection and cellular damage by activating inflammatory caspase-1 (3). More specifically, after assembly and activation of the NLRP3 inflammasome, procaspase-1 is hydrolyzed into an active form, and activated caspase-1 induces maturation of the proinflammatory factors IL-1β and IL-18 for their secretion out of the cell, inducing an inflammatory cascade. The NLRP-3 inflammasome can be activated in multiple ways, such as the binding of adenosine triphosphate (ATP) to P2X7 receptors (4). Moreover, previous studies have shown that the NLRP3/Caspase-1 inflammasome pathway can also be activated by lipopolysaccharide (LPS) through a process involving ATP-activated P2X7 receptors (5). However, the specific mechanism of NLRP3/Caspase-1 inflammasome pathway activation remains unclear.

Studies have shown that the activated NLRP3 inflammasome induces podocyte injury or disappearance, proteinuria, and even glomerulosclerosis by inducing the release of inflammatory factors and abnormal expression of specific structural proteins, eventually leading to the occurrence and development of podocyte injury-related kidney disease (6). Moreover, inhibition of NLRP3 inflammasome activation was reported to increase the expression of podocin and nephrin and decrease the expression of desmin, a podocyte injury-related protein, alleviating podocyte injury (7, 8). Therefore, the NLRP3 inflammasome plays an important role in the pathogenesis of HBV-GN, and treatments targeting signaling pathways upstream and downstream of NLRP3 may be novel strategies for clinical treatment.

In the present work, we aimed to investigate whether HBx promotes podocyte pyroptosis by activating the NLRP3 inflammasome. In addition, we explored the mechanism of oxidative stress in the pathogenesis of HBV-GN.

## Materials and Methods

*Cell line, culture, and treatment*

Podocytes are terminally differentiated visceral epithelial cells that are pivotal for glomerular function. They enwrap the glomerular capillaries by interdigitating foot processes that build up a slit diaphragm for ultrafiltration of the blood (9, 10).

A kidney podocyte cell line (cat# GD-C8618339) was cultured in DMEM containing 4.5 mM glucose, 10% FBS, 100 U/ml penicillin, and 100% streptomycin at 37 °C in a humidified atmosphere of 5% CO_2_ and 95% air. The medium was changed daily. Cells used for assays were detached from the medium with a solution of 0.25% trypsin and 0.02% EDTA. Before treatment, 80–85% confluent cells were incubated with serum-free medium for 12 hr to arrest the cell cycle for synchronous growth.

*Lentivirus and siRNA transfection*

HBx overexpression lentiviruses, negative control (NC) lentiviruses, and NLRP3 siRNA were synthesized by GenePharma (Shanghai, China). For cell transduction, kidney podocyte cells at a density of 50,000 cells per well were seeded into 24-well plates and transfected according to the manufacturer’s instructions. Subsequent cell culture was carried out as described above. We refer to the cells transfected with HBx lentivirus as OE-HBx cells and the cells transfected with NG lentivirus as OE-NC cells. The group of cells transfected with HBx lentivirus and NLRP3 siRNA is referred to as OE-HBx+si-NLRP3 cells. We used a reactive oxygen species (ROS) inhibitor to define the function of HBx, and we named this group the OE-HBx+ROS inhibitor group.

*Hoechst 33342 staining*

Morphological changes due to pyroptosis in the cells were observed by staining the nuclei with Hoechst 33342 (C0030, Solarbio, Beijing, China). Hoechst 33342 was added to cells transfected with different vectors, and the cells were cultured in an incubator for 20–30 min. The medium was discarded, and the cells were washed with medium 2–3 times. Then, the cells were immediately observed and imaged under a fluorescence microscope (Olympus, Tokyo, Japan), and the concentrated stained cells in 5 fields were randomly counted.

*Caspase-1 activity assay*

Caspase-1 activity was measured with the Caspase-1 Activity Assay Kit (Sigma, St. Louis, MO, USA) according to the manufacturer’s protocol. Briefly, 2 × 10^6^ cells were centrifuged at 1200 rpm for 5 min and washed two times with PBS (pH 7.4) at 4 °C. The cells were resuspended in 50 μl of cell lysis buffer, and all subsequent steps were performed on ice. The protein concentration was measured using a Micro BCA kit. Each 50-μl sample of the cell extract (containing 100 µg of protein) was combined with an equal volume of 2 × reaction buffer in a microplate, and 5 μl of peptide (a substrate of caspase-8) was added. After overnight incubation in the dark at 37 °C, the absorbance of the samples at 405 nm was examined by a microplate reader. Caspase-1 activity was calculated as the ratio of the absorbance of the treated group to that of the control group.

*Ultrastructural evaluation*

For electron microscopy investigations, at least 12 biopsies with binucleated podocytes in each renal entity were analyzed as ultrathin sections using scanning transmission electron microscopy (STEM) with a Zeiss Sigma electron microscope (Zeiss, Oberkochen, Germany) at 5000–10000× magnification. Measurement of the foot process width in podocytes that were clearly mononucleated, lobulated, potentially binucleated, binucleated, or multinucleated was performed using the ZEN software (Zeiss).

*LDH release assay*

Cell culture supernatants were collected, and LDH activity was detected using an LDH ELISA Assay Kit (Nanjing Jiancheng Biology Engineering Institute, Nanjing, Jiangsu, China). Briefly, 25 μl of cell supernatant and 25 μl of substrate were mixed and incubated at 37 °C for 15 min. Then, 25 μl of 2,4-dinitrophenylhydrazine was added to the samples and incubated at 37 °C for 15 min. Finally, 250 μl of a 0.4 mol/L NaOH solution was added and incubated at room temperature for 5 min. The absorbance at 450 nm was measured on a spectrophotometric microplate reader.

*Measurement of ROS*

A Reactive Oxygen Species Assay Kit (CA1410, Solarbio, Beijing, China) was used to quantitatively analyze oxidation by measuring the modification of cell-permeable 2,7-dichlorodihydrofluorescein diacetic acid (DCFH-DA) to fluorescent DCF by flow cytometry. DCFH-DA was diluted to 10 μmol/l in a serum-free medium based on the manufacturer’s instructions, and cells transfected with different vectors were resuspended in the diluted reagent and incubated in an incubator for 20 min. Subsequently, the serum-free medium was washed three times, and fluorescence intensity was detected by flow cytometry.

*RNA isolation and RT–PCR*

The detailed protocol was used to isolate intimal RNA from the aorta. TRIzol reagent (Invitrogen, CA, USA) was applied to extract total RNA from the intima and human aortic endothelial cells (HAECs). A High-Capacity cDNA Reverse Transcription Kit (Applied Biosystems, Foster City, CA, USA) was used for reverse transcription of extracted RNA into cDNA. First-strand cDNA was amplified using SYBR Green I to quantify the relative expression of mRNA on an ABI 7500 Fast Real-Time PCR system (Applied Biosystems, USA). After amplification, the threshold cycle (Ct) was determined, and relative mRNA levels were calculated based on the 2^−Ct^ method. GAPDH was used as an internal control for data normalization. The following primer sequences were used:

HBx forward primer:

5’-GCGAATTCATGGCTGCTAGGGTGTGCT-3’

HBx reverse primer: 

5’-ATCTCGAGTTAGGCAGAGGTGAAAAAGTTGC-3’

NLRP3 forward primer: 

5’-CACCTGTTGTGCAATCTGAAG-3’

NLRP3 reverse primer: 

5’-GCAAGATCCTGACAACATGC-3’

ASC forward primer: 5’-AGGCCTGCACTTTATAGACC-3’

ASC reverse primer: 5’-GCTGGTGTGAAACTGAAGAG-3’

Caspase-1 forward primer: 

5’-CCTTAATATGCAAGACTCTCAAGGA-3’

Caspase-1 reverse primer: 

5’-TAAGCTGGGTTGTCCTGCACT-3’

IL-1β forward primer: 

5’-TACCTGTCCTGCGTGTTGAA-3’

IL-1β reverse primer: 

5’-TCTTTGGGTAATTTTTGGGATCT-3’

IL-18 forward primer: 5’-TGCATCAACTTTGTGGCAAT-3’

IL-18 reverse primer: 5’-ATAGAGGCCGATTTCCTTGG-3’

GAPDH forward primer: 

5’-AAGGTCGGAGTCAACGGATTT-3’

GAPDH reverse primer: 5’- AGATGATGACCCTTTTGGCTC-3’

*Flow cytometry analysis*

The percentage of pyroptotic cells was determined by flow cytometry using a pyroptosis flow detection kit. Kidney podocytes were plated into 6-well plates (5×10^5^/ml) and cultured overnight. After transfection with different plasmids, pyroptosis was analyzed by flow cytometry. The cells were then digested, centrifuged, washed three times with PBS, and suspended in 500 µl of binding buffer (1×), forming a cell suspension at a concentration of ~ 1x10^6^ cells/ml. The stain was added according to the instructions and incubated while protected from light. Cells were immediately tested using flow cytometry (within 1 hr), and data analysis was performed with the software. Pyroptotic cells were quantified as the sum of the numbers of cells in quadrants Q2 and Q4.

*Western blot analysis*

Total protein was extracted from endothelial cells using procedures described in detail elsewhere. The protein concentrations were determined with a BCA Protein Assay Kit (Bio-Rad, Mississauga, ON, Canada). Equal amounts of protein lysates were separated by SDS–PAGE and transferred onto nitrocellulose membranes, followed by blocking with 5% skimmed milk at room temperature for 2 hr. Subsequently, the membranes were incubated with primary antibodies against NLRP-3 (Proteintech, Chicago, USA, 1:1000, Cat. No.: 19771-1- AP), ASC (Santa Cruz, USA, 1:500, Cat. No.: sc-22514-R), Caspase-1 (Proteintech, Chicago, USA, 1:1000, Cat. No.: 22915-1-AP), IL-1β (ABclonal, Boston, USA, 1:1000, Cat. No.: A1112), IL-18 (ABclonal, Boston, USA, 1:1000, Cat. No.: A1115), or GAPDH (Proteintech, Chicago, USA,1:2000, Cat. No: 60004-1-lg) at 4 °C overnight. After washing with PBST three times, the membranes were incubated with the fluorescence-conjugated anti-rabbit IgG secondary antibody (1:10,000) for 1 hr. Western blot bands were examined and analyzed by an Odyssey Imaging System (LI-COR, Inc., Lincoln, NE, USA).

*Immunofluorescence staining*

Cells in 6-well plates were fixed with 4% paraformaldehyde (PFA) at room temperature for 30 min, incubated in PBS containing 0.4% Triton X-100 for 10 min, and then blocked with 2% bovine serum albumin (BSA) at 37 °C for 60 min. After the blocking step, the cells were incubated with primary antibodies against vimentin (1:100, Boster, Wuhan, China) and keratin (1:100, Boster) at 4 °C overnight; PBS was used as a negative control. Then, the cells were washed with PBS and incubated with anti-mouse IgG Alexa Fluor 488 or anti-rabbit IgG Alexa Fluor 594 (1:1000) (Life Technologies, Paisley, UK) at room temperature for 1 hr. Glass cover slides were applied using an installation medium with DAPI dye (Vector Labs, Peterborough, UK) and prepared for imaging under a fluorescence microscope (Olympus, Tokyo, Japan).

*Data analysis*

The data are expressed as the mean ± SD. GraphPad Prism 8.0 software package was used to process the data. For comparisons between two groups, the unpaired t-test was performed; for comparisons among multiple groups, one-way analysis of variance (ANOVA) was performed. *P*<0.05 was considered to indicate statistical significance.

## Results

***HBx affects cell pyroptosis through NLRP3 and ROS***

**Figure 1:**
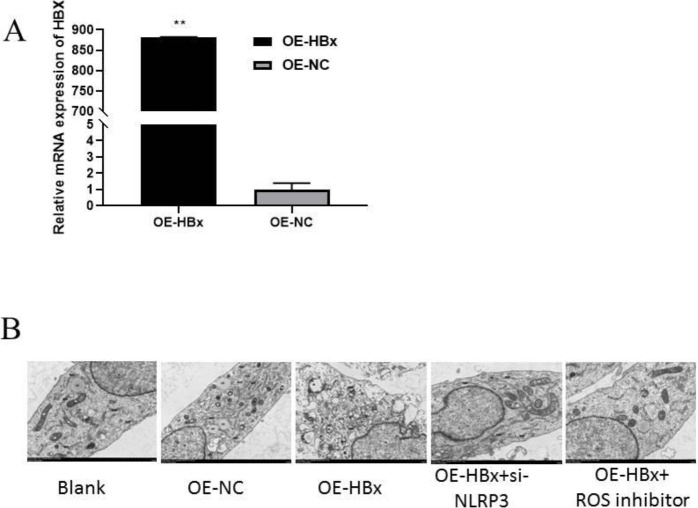
HBx affects cell pyroptosis through NLRP3 and ROS. (A) HBx expression was measured by RT-PCR. (B) Electron microscopy images showing that the morphology of podocytes changed after HBx overexpression, as the organelles were severely damaged, but no obvious pyroptosis was observed after NLRP3 knockdown. OE, overexpression; NC, negative control; siRNA, small interfering RNA; ROS, reactive oxygen species; N, nucleus; M, mitochondrion; RER, rough endoplasmic reticulum; LD, lipid droplet

To verify whether HBx affects the pyroptosis of renal podocytes, the HBx gene was overexpressed in normal renal podocytes, and the HBx mRNA expression level was detected by qPCR (Figure 1A). Then, changes in cell morphology were examined by electron microscopy (Figure 1B). As revealed by electron microscopy, no significant difference in pyroptosis between cells from blank and NC groups was observed, whereas cells from the HBx-overexpression group showed irregular nuclei, severely swollen mitochondria, vacuolized and severely edematous cytoplasm, and broken and discontinuous cell membranes. However, the cells in the NLRP3-knockdown group and ROS-inhibition group tended to be closer to normal than those in the HBx-overexpression group (Figure 1B). These findings demonstrated that NLRP3 and ROS play crucial roles in the process of HBx-induced pyroptosis.

***HBx regulates pyroptosis through regulation of the oxidative stress pathway***

**Figure 2:**
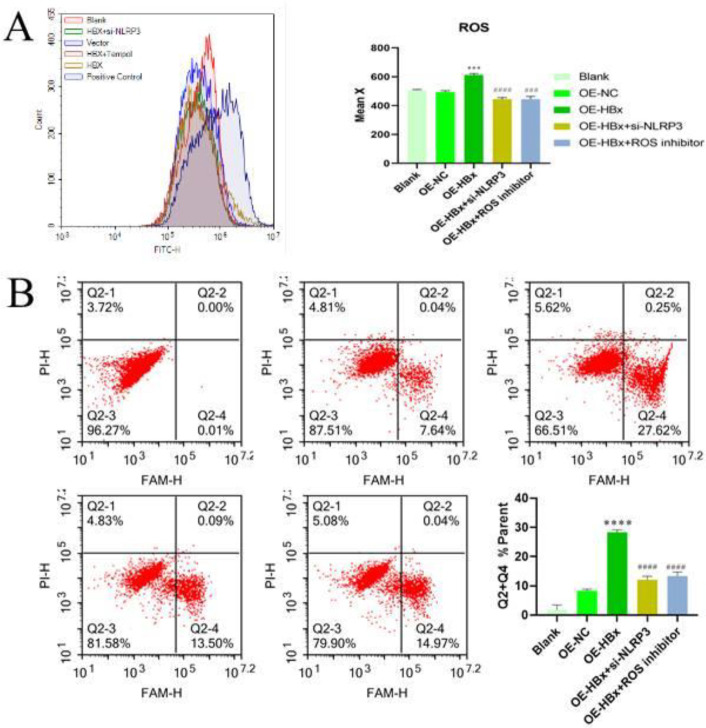
HBx regulates pyroptosis through regulation of the oxidative stress pathway. (A) Flow cytometric detection showed that HBx overexpression promoted ROS generation. Knockdown of NLRP3 inhibited ROS generation (n=3). (B) Flow cytometry assays showed that HBx overexpression promoted pyroptosis, which was reversed by knockdown of NLRP3 or the addition of an ROS inhibitor (n=3). (***P<0.001 and ****P<0.0001 compared with OE-NC; ### *P*<0.001 and #### *P*<0.0001 compared with OE-HBx). OE, overexpression; NC, negative control; siRNA, small interfering RNA; ROS, reactive oxygen species

To further verify the mechanism of HBx-NLRP3 in pyroptosis, flow cytometry was used to detect the levels of ROS in different groups. The data showed that ROS levels were obviously increased after HBx overexpression compared with those in the control group, but the ROS level decreased with NLRP3 knockdown (*P*<0.05). This demonstrated that oxidative stress may play a very important role in the process of pyroptosis induced by HBx (Figure 2A). A pyroptosis detection kit and flow cytometry were subsequently used to detect pyroptosis in each group. The sum of the numbers of cells in quadrants 2 and 4 in the flow cytometry data was counted as the number of pyroptotic cells. HBx overexpression significantly increased the proportion of cells undergoing pyroptosis, which was also significantly inhibited in both the NLRP3-knockdown and ROS-inhibitor groups (*P*<0.05) (Figure 4B). 2B). All subsequent experiments after this point were performed with the addition of an ROS inhibitor to verify the role of the ROS pathway in regulation of pyroptosis.

***HBx induces pyroptosis by regulating Caspase-1 enzyme activity and LDH expression via the ROS-NLRP3 pathway***

**Figure 3:**
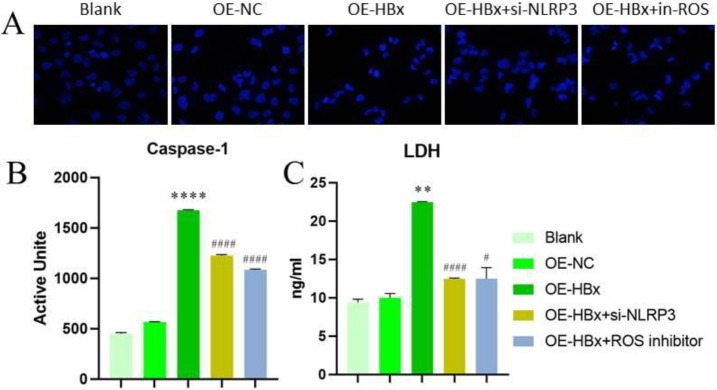
HBx induces pyroptosis by regulating Caspase-1 enzyme activity and LDH expression via the ROS-NLRP3 pathway. (A) Hoechst 33342 staining showed that pyroptotic cells in the HBx-overexpression group clearly exhibited pyknosis, which could be reversed by down-regulation of NLRP3 or ROS inhibition. (B) (C) Results from caspase-1 enzyme activity as assay and ELISA showed that caspase-1 enzyme activity in pyroptotic cells was significantly increased and that LDH release was significantly increased in the HBx-overexpression group; both of these changes were reversed by down-regulation of NLRP3 or ROS inhibition (n=3). (** *P*<0.01 and ****P<0.0001 compared with OE-NC; #*P*<0.05 and #### *P*<0.0001 compared with OE-HBx). OE, overexpression; NC, negative control; siRNA, small interfering RNA; ROS, reactive oxygen species

When cells undergo pyroptosis, the nucleus condenses to varying degrees, giving a dense appearance under Hoechst 33342 staining. Hoechst 33342 staining showed that the proportion of cells with nuclear condensation in the HBx-overexpression group was significantly higher than that in the control group, but this change was reversed by the knockdown of NLRP3 or the addition of an ROS inhibitor (Figure 3A). We also assayed the enzymatic activity of caspase-1, which was significantly increased in the HBx-overexpression group; similarly, inhibition of NLRP3 and ROS expression reduced caspase-1 enzyme activity (*P*<0.05) (Figure 3B). ELISA was then used to detect LDH expression, which revealed the same trend, as shown in Figure 3C.

***Effect of NLRP3 and ROS on HBx-induced inflammasome pathway activation***

**Figure 4:**
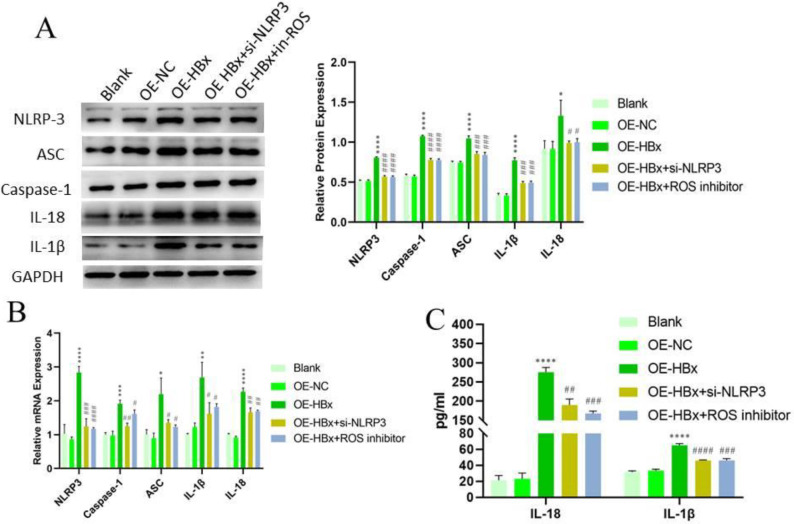
Effect of NLRP3 and ROS on HBx-induced inflammasome pathway activation. (A) Protein expression was determined by Western blotting, and GAPDH was used as a control. (n=3) (B) (C) RT–PCR was used to analyze the mRNA expression levels of NLRP3, ASC, caspase-1, IL-18, and IL-1β (n=3). (D) ELISA was used to detect cytokine release (n=3). (*P<0.05, **P<0.01, ***P<0.001, and ****P<0.0001 compared with OE-NC; #*P*<0.05, ##*P*<0.01, ###*P*<0.001, and ####*P*<0.0001 compared with OE-HBx). OE, overexpression; NC, negative control; siRNA, small interfering RNA; ROS, reactive oxygen species; mRNA, messenger RNA; RT–PCR, reverse transcription-polymerase chain reaction

RT–PCR and Western blotting were used to detect the expression of genes related to the inflammasome pathway upon HBx induction. When HBx was overexpressed, intracellular inflammasome pathway-related genes, such as NLRP3, ASC, caspase-1, IL-1β, and IL-18, were synchronously up-regulated. Down-regulation of NLRP3 expression and ROS inhibition decreased the expression levels of these genes (*P*<0.05) (Figures 4A and 4B). ELISA was also used to detect IL-1β and IL-18 release. The results showed that HBx overexpression increased the release of these cytokines. However, the levels of these cytokines were decreased by down-regulation of NLRP3 and inhibition of ROS.

***Detection of podocyte protein expression by immunofluorescence staining***

**Figure 5:**
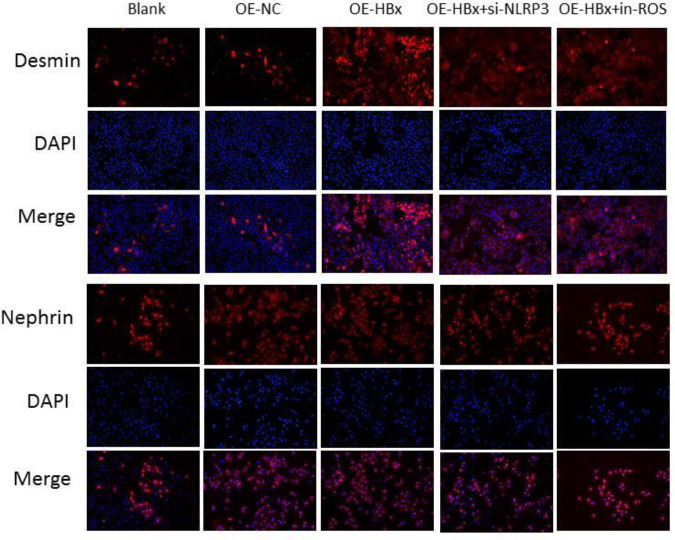
Desmin and Nephrin expression was detected by immunofluorescence. Desmin expression was positively correlated with HBx expression, and Nephrin expression was negatively correlated with HBx expression. OE, overexpression; NC, negative control; siRNA, small interfering RNA; ROS, reactive oxygen species; in-ROS, ROS inhibitor; DAPI, 4’, 6-diamidino-2-phenylindole

Immunofluorescence staining was used to detect the expression profiles of Desmin and Nephrin in renal podocytes. The staining results showed that the protein expression of Desmin, a marker of podocyte injury, was significantly increased when HBx was overexpressed, and this change was reversed upon decreased NLRP3 expression and ROS inhibition. Defects in nephrin integrity were reported to reduce podocyte viability in hyperglycemia-mediated podocyte injury (11). As expected, the expression of HBx was inversely correlated with the expression of Nephrin (Figure 5A).

## Discussion

In the present study, we explored the specific regulatory mechanism by which HBx induces pyroptosis in renal podocytes, which fully confirmed the pathological effects of the NLRP3 inflammasome. In addition, we found that the ROS/NLRP3 pathway is closely associated with the development of podocyte pyroptosis in HBV-GN.

Hepatitis B is one of the most prevalent infectious diseases in China, and chronic inflammation of the liver or other organs caused by a high HBV load can lead to organ fibrosis, cell necrosis, apoptosis, and ultimately organ failure (12). HBV-GN is one of the most common secondary kidney diseases in China (13). Moreover, membranous nephropathy is the most common pathological type of HBV-GN, and the course of this disease progressively contributes to podocyte injury, which in turn accelerates the progression of kidney disease. At present, HBV-GN is mainly treated with antiviral therapy, but these therapies are greatly limited in their ability to improve the glomerular filtration rate (14). Xie *et al*. found that HBx activates the NLRP3/ROS pathway in normal hepatocytes, providing novel insights into therapies for HBV-induced hepatitis [15). Therefore, further exploration of the pathological process of HBV-GN is greatly needed to help improve HBV-GN treatment.

 Previous studies have shown that  expression of HBV proteins (particularly the HBx protein) plays a critical role in HBV-GN (16, 17). However, in addition to hepatocellular carcinomas (18), HBx may play an important role in inflammation in response to HBV proteins (19-20). Therefore, the HBx gene was overexpressed to investigate the mechanism of podocyte pyroptosis in HBV-GN. Our results demonstrated that the HBx gene plays a pivotal role in podocyte injury in HBV-GN, consistent with the findings of *previous work from* He *et al*. (21).

The activation of inflammasome-dependent NLRP3 requires two steps (22). First, NF-κB activators, such as LPS, bacteria, and viruses, prime the NLRP3 inflammasome. Next, active caspase-1 activates pro-IL-1β and pro-IL18 into IL-1β and IL-18, respectively, and cleaves gasdermin-D (GSDMD), inducing pyroptotic cell death. The activated NLRP3 inflammasome carries out various functions in the body. It is not only involved in numerous pathological processes, such as infection and autoimmunity but can also defend against external invaders, for example, in antitumor immunity (23, 24). Additionally, high NLRP3, GSDMD, caspase-1, IL-1β, and IL-18 expression levels were found to be positively correlated with inflammatory activity in the liver in patients with active chronic HBV (25), which further illustrated that the detection of these indicators is of great interest. ASC, a constituent of the NLRP3 inflammasome, has been shown to induce acute inflammatory reactions in mice (26). In recent years, some kidney diseases that can lead to end-stage renal disease (ESRD), including acute kidney injury (AKI), diabetic kidney disease, and renal fibrosis, have been confirmed to be commonly induced by a certain level of inflammation (27). These intersecting findings indicate that the NLRP3 inflammasome may play a role in the occurrence and progression of kidney diseases. However, the molecular mechanism of podocyte pyroptosis among HBx-induced processes needs to be further elucidated. In the present study, we knocked down NLRP3 under HBx induction and used RT–PCR and Western blotting to detect the expression of genes related to the NLRP3 inflammasome pathway. The results demonstrated increased levels of pyroptotic markers stimulated by HBx overexpression, which were reversed by NLRP3 knockdown *in vitro*. Hence, NLRP3 regulates HBx-induced podocyte pyroptosis.

The NLRP3 inflammasome can be activated by a diverse array of internal and external environmental factors, among which mitochondrial damage is a potential research hotspot. Moreover, the ROS/NLRP3 pathway is widely activated in multiple diseases. In addition to diabetes, ischemic stroke, and acetaminophen (APAP)-induced liver disease (28-30), the ROS/NLRP3 pathway plays a major role in some kidney diseases. For example, the alternative polyadenylation trans-factor FIP1 contributes to AKI-chronic kidney disease (CKD) transition via the ROS-NLRP3 axis (31). Moreover, NADPH oxidase (NOX) and its derived products control ROS-mediated triggering of inflammasome activation during hyperhomocysteinemia (32-34). Furthermore, several studies have reached similar conclusions in lupus nephritis (LN) (35) and podocyte injury(36). In light of these studies, we hypothesized that the activation of NLRP3 under HBx induction is associated with the production of ROS. Thus, flow cytometry and an ROS assay kit were used to detect the levels of ROS in different groups. As expected, the increase in ROS level under HBx induction was reversed with NLRP3 knockdown, which indicated that mitochondrial ROS play an important role in HBx-induced pyroptosis.

Further studies on the pathways both upstream and downstream of the NLRP3 inflammasome will provide new therapeutic targets for multiple kidney diseases. A recent study showed that ibudilast effectively attenuates AKI by inhibiting activation of the NLRP3 inflammasome through TLR4-mediated NF-κB pathways (37). Similarly, some clinical drugs inhibit ROS production and regulate NLRP3 function to exert their clinical efficacy. For example, osthole and dihydroquercetin play roles protecting against progressive IgA nephropathy and diabetic nephropathy, respectively (38-39). Additionally, the protective effect of N-acetylcysteine (NAC) in diabetes-related ocular surface damage may be attributed to inhibition of the ROS/NLRP3/Caspase-1/IL-1β pathway (40). Although elimination of many ROS has been demonstrated to be beneficial in experimental models, caution is required due to the potential hazards of inhibiting physiological ROS (41). In the present study, we demonstrated that HBx induces pyroptosis via the ROS-NLRP3 pathway, which may provide an effective potential therapeutic approach for HBV-GN.

While it provides important new findings, the limitations of the current work are also noted. First, only one human normal kidney cell line was used in this study. Other normal renal cell lines, such as tubular epithelial cells and glomerular epithelial cells, need to be evaluated. In addition, the present study did not include any animal experiments, which may provide relevant and necessary insights. Nevertheless, our study may provide new insights into the pathogenesis of HBV-induced nephritis.

## Conclusion

Based on our findings, activation of the NLRP3 inflammasome and podocyte injury is causally related. The results of the present study demonstrate for the first time that HBx induces podocyte pyroptosis via the ROS/NLRP3 pathway in HBV-GN. As a new target for prevention of podocyte injury, the NLRP3 inflammasome is of great significance for prevention and treatment of kidney disease. With additional related research, these findings may provide a new method for the prevention and treatment of HBV-GN.

## Authors’ Contributions

YY and HD wrote the main manuscript and performed the study. JS and BL collected the study data. YC, MF, and XY finished the formal analysis and investigation. WJ prepared Figures 1–4 and approved the final version of the manuscript. All authors reviewed the manuscript.

## Data Avaiability Statment

Not applicable.

## Conflicts of Interest

No conflicts of interest have been declared by the authors.
